# Splice-switching antisense oligonucleotides for pediatric neurological disorders

**DOI:** 10.3389/fnmol.2024.1412964

**Published:** 2024-07-25

**Authors:** Xiaochang Zhang

**Affiliations:** Department of Human Genetics, The Neuroscience Institute, University of Chicago, Chicago, IL, United States

**Keywords:** ASO, SSO, neurodevelopmental disorder, epilepsy, autism, alternative splicing, nonsense-mediated mRNA decay, Syngap1

## Abstract

Pediatric neurological disorders are frequently devastating and present unmet needs for effective medicine. The successful treatment of spinal muscular atrophy with splice-switching antisense oligonucleotides (SSO) indicates a feasible path to targeting neurological disorders by redirecting pre-mRNA splicing. One direct outcome is the development of SSOs to treat haploinsufficient disorders by targeting naturally occurring non-productive splice isoforms. The development of personalized SSO treatment further inspired the therapeutic exploration of rare diseases. This review will discuss the recent advances that utilize SSOs to treat pediatric neurological disorders.

## Introduction

Over the last two decades, causal variants for pediatric neurological disorders have been increasingly uncovered by high-throughput DNA sequencing. Many clinically comparable disease symptoms, such as developmental and epileptic encephalopathy (DEE), turn out to be caused by mutations in dozens of genes that have different biological functions and pathophysiology. Consequently, human diseases are increasingly classified based on their molecular causes and clinical presentations. Such accumulating genetic evidence offers unique opportunities to develop gene- or variant-specific treatments in addition to generic symptom-oriented drugs. Precision medicine strategies for neurological disorders, such as gene replacement therapy, genome editing, and splicing modulation, have been actively explored (Deverman et al., 2018; Nussbacher et al., 2019; Raguram et al., 2022). Antisense oligonucleotides (ASO) represent one type of such therapeutic means and have shown promising clinical outcomes for spinal muscular atrophy (SMA), Duchenne muscular dystrophy (DMD), and Amyotrophic Lateral Sclerosis (ALS), among other ongoing clinical and preclinical studies (Rinaldi and Wood, 2018).

ASOs are modified short nucleotides that bind to pre-mRNA through Watson-Crick base pairing (Kole et al., 2012). ASOs can be used as steric blockers to intervene in processes such as splicing and protein translation, or as gapmers to promote RNase H1-mediated target mRNA degradation. The nucleobases and the backbone are modified to resist nuclease degradation, enhance the target binding, and boost cellular intake. ASO modifications, such as 2’-O-methoxyethyl-modified (MOE) nucleotides with phosphorothioate (PS) backbone, have been clinically tested and proven to be generally tolerated (Egli and Manoharan, 2023). Various modifications have been developed to enhance efficacy and decrease toxicity. Splice-switching oligonucleotides (SSO) are a specific category of ASOs that bind to pre-mRNA as steric blockers and redirect splicing. SSOs have been successfully developed to treat SMA and DMD (Voit et al., 2014; Finkel et al., 2017). ASO gapmers have been recently approved by the FDA to treat SOD1 ALS. This review focuses on the progress of SSOs in targeting pediatric neurological conditions.

Most human protein-coding genes are split by introns, which are spliced out by the spliceosome (Berget et al., 1977; Chow et al., 1977). Introns are collectively defined by their 5′ splice donor site (5’SS), 3′ acceptor site (3’SS), the branchpoint adenosine, the poly-pyrimidine tract upstream of the 3’SS, and other regulatory sequences. Pre-mRNA splicing allows the reshuffle of different exons (Gilbert, 1978), and RNA-seq analyses showed that over 95% of intron-containing human genes undergo alternative splicing (AS) to generate multiple mRNA isoforms (Pan et al., 2008; Wang et al., 2008). Alternative splicing can lead to skipped exons (SE), alternative 5′ splice site (A5SS), alternative 3′ splice site (A3SS), mutually exclusive exons (MXE), and retained introns (RI) (Graveley, 2001). Alternative splicing can happen in species-, tissue- and cell-type-specific manners (Barbosa-Morais et al., 2012; Merkin et al., 2012; Feng et al., 2021). Alternative splicing is prevalent in the brain, and recent long-read sequencing analyses have uncovered coordinated splicing of distant exons (Gupta et al., 2018; Yang et al., 2023; Zhang et al., 2023). Alternative splicing is modulated by intronic and exonic cis-regulatory sequences and their associated RNA-binding proteins (Black, 2003; Wang and Burge, 2008; Barash et al., 2010; Xiong et al., 2015; Bao et al., 2019; Van Nostrand et al., 2020). The natural occurrence of alternative splicing and the identification of splicing enhancers/suppressors indicate that re-directing splicing holds its own dimension for gene regulation and therapeutic intervention.

About 10% of exonic human mutations are estimated to cause diseases by disrupting pre-mRNA splicing (Soemedi et al., 2017). While whole-exome sequencing detects exonic and splice site mutations for genetically defined disorders, integrating transcriptome and whole-genome analysis uncovers more causal intronic splicing mutations (Cummings et al., 2017; Kim et al., 2023). These splicing mutations frequently introduce aberrant splice sites that lead to loss-of-function or hypomorphic alleles. Disease-causing splicing variants can be suppressed to treat human diseases. Redirecting splicing can also lead to beneficial effects by (1) bypassing nonessential inframe exons that carry pathogenic mutations, (2) bypassing an additional exon to correct the reading frame, and (3) redirecting alternative splicing to promote functional isoform production.

SSOs bind to pre-mRNA through Watson-Crick base pairing and redirect pre-mRNA splicing (Kole et al., 2012; Centa and Hastings, 2022). The SSO binding sites are frequently splicing enhancers or suppressors, and the double-stranded SSO-pre-mRNA can block RNA–RNA or RNA-protein interactions that modulate splice site usage. Since the success of SSOs in treating DMD and SMA (Hua et al., 2011; Finkel et al., 2017), redirecting pre-mRNA splicing has been increasingly recognized as a powerful therapeutic strategy to treat neurological disorders (Hill and Meisler, 2021; Nikom and Zheng, 2023). The sequence flexibility and the clinically proven chemistry have made SSO a fast-growing platform for personalized medicine. The development of the SSO drug Milasen for a girl named Mila is inspirational, and the approach displayed promising progress toward previously undruggable targets and rare mutations (Kim et al., 2019, 2023). This review focuses on recently reported SSO strategies targeting pediatric neurological conditions and the value of genetic tools.

**Figure 1:**
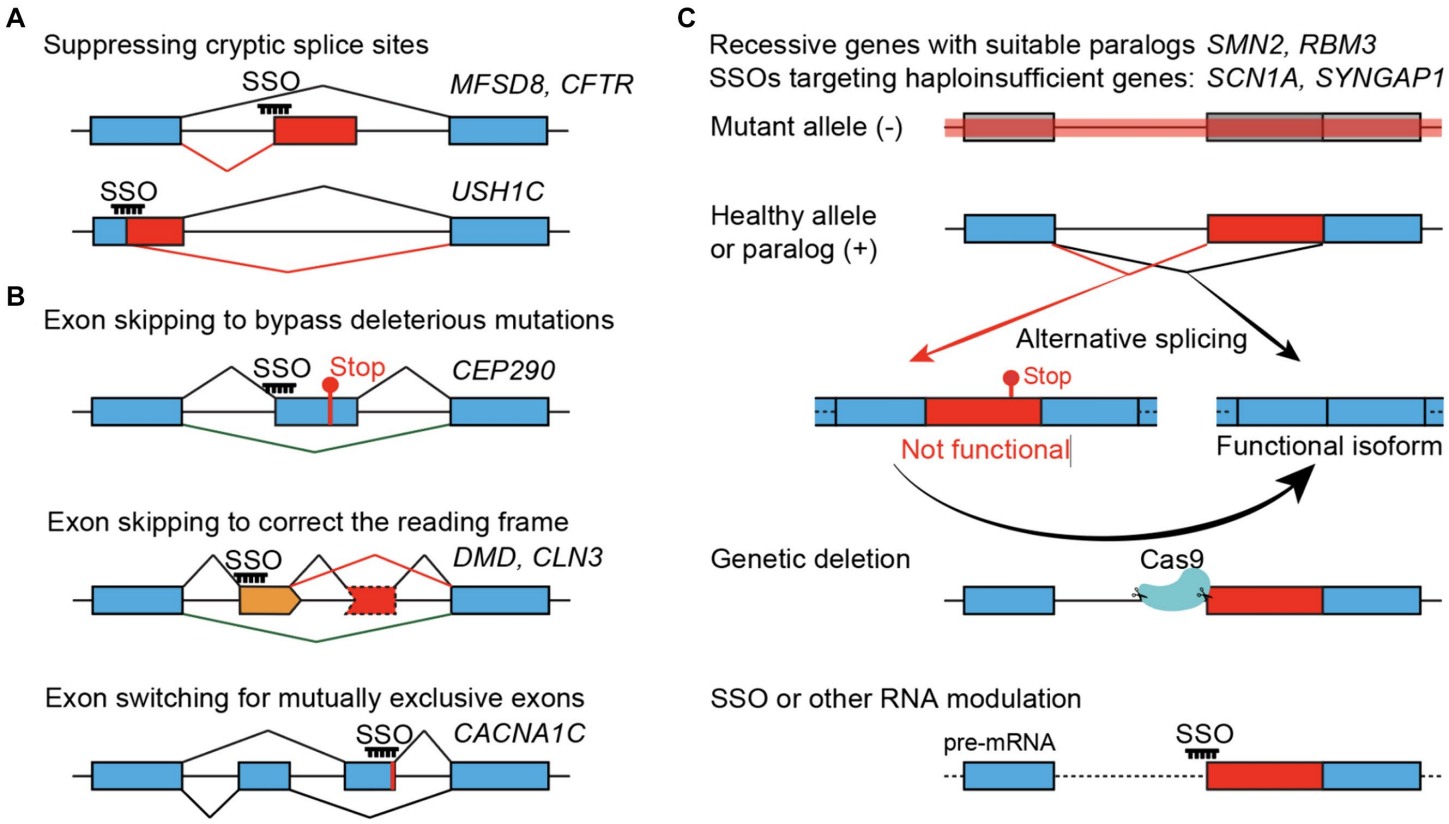
SSO-mediated therapeutic strategies. **(A)** Variant-specific SSOs suppress the gain of cryptic splice sites in the introns (top) or exons (bottom). **(B)** Exon-specific SSOs. Bypassing a non-essential exon that carries pathogenic mutations (top), skipping an additional non-essential exon (orange) to correct the translational reading frame (middle), or switching for a functional mutually exclusive exon (bottom). **(C)** Gene-specific SSOs treating recessive or haploinsufficient conditions by converting naturally occurring non-functional (or unstable) splice isoforms to functional isoforms, using *SYNGAP1* as an example. Genetic suppression of non-productive splicing, mimicking the maximal and constant effect of an SSO, can provide *in vivo* evidence about the neurological and organismal functions of the non-productive isoform, to what extent the protein level can be restored, and whether it can rescue phenotypes associated with loss-of-function alleles.

SSOs can promote either exon skipping or inclusion. With a focus on pediatric neurological conditions, recently reported SSO strategies generally fall into three main categories (Figure 1). The most straightforward application of SSOs would be suppressing an abnormal splice site introduced by a specific mutation – a variant-specific SSO (Figure 1A). SSOs have also been developed to skip a nonessential exon that is either inframe and carries deleterious mutations or correct the reading frame caused by frameshift mutations – an exon-specific SSO (Figure 1B). Lastly, SSOs can increase protein expression through a paralog and rescue recessive diseases or boost protein expression from the wild-type allele and rescue haploinsufficiency – such SSOs are independent of mutations and can be considered gene-specific SSOs (Figure 1C). While variant- and exon-specific SSOs play prominent roles in personalized medicine, a gene-specific SSO can be used to treat patients carrying mutations across the same gene. SSO-mediated therapy, or treatments for genetic disorders in general, would be more effective when used for sooner intervention in disease progression than later. Thus, early genetic diagnosis-aided treatment before the existence of irreversible disease presentations, as shown by a recent study (Kim et al., 2023), appears to be a promising practice.

### SSOs for recessive diseases

Recessive diseases frequently involve loss-of-function alleles, and several SSO-based therapeutic strategies have been reported (Figure 1). SSO can promote the inclusion or exclusion of specific exons. Thus, it is straightforward to use SSOs to suppress undesired exons, such as abnormal/cryptic splice sites. SSOs can also block splicing silencers and promote exon inclusion to make functional proteins, such as the *SMN2* case below.

Spinal muscular atrophy (SMA) is a motor neuron disorder caused by recessive loss-of-function mutations in *SMN1* (Lefebvre et al., 1995). The loss of spinal cord motor neurons in SMA patients leads to muscle weakness and atrophy, and the disease presentations fall into different clinical categories based on the age of onset and the severity of symptoms. Type 1 SMA, with the disease onset by 6 months of age and an expected life shorter than 2 years, is the most severe form and affects about 50% of all cases. *SMN2* is a hominid-specific duplication of *SMN1*, and increased *SMN2* copy numbers are inversely correlated with SMA severity (Rochette et al., 2001; Calucho et al., 2018). Compared to *SMN1*, *SMN2* carries a single synonymous C-to-T change in exon 7 that causes 90% of *SMN2* mRNA to skip exon 7 and encode an unstable protein (Monani et al., 1999). The exon7-included *SMN2* mRNA encodes an identical protein to SMN1. Multiple strategies, including SSOs and splicing modulatory small molecules, have been developed to promote *SMN2* exon 7 inclusion and treat SMA (Hua et al., 2010). The FDA-approved SSO Spinraza/nusinersen consists of 18 2’-MOE nucleotides with a PS backbone, binds to *SMN2* intron7, and promotes the inclusion of *SMN2* exon7 (Hua et al., 2011). Nusinersen has been shown to significantly improve the motor conditions and life expectancy of SMA patients (Finkel et al., 2017; Mercuri et al., 2018).

A straightforward application of SSOs would be suppressing abnormal/cryptic splice sites introduced by pathogenic mutations (Figure 1A). This strategy has been explored to treat multiple diseases, such as the *USH1C* Usher syndrome (Lentz et al., 2013). Autosomal recessive *USH1C* mutations cause type 1 Usher syndrome concerning congenital sensorineural deafness, vestibular dysfunction, and blindness (Verpy et al., 2000). The *USH1C* c.216G > A creates a cryptic 5′ splice site in exon 3, and an SSO covering the mutation and cryptic splice site significantly corrected the splicing error (Lentz et al., 2013). Remarkably, a single-dose SSO injection in the neonatal *Ush1c* c.216AA mice rescued abnormalities of cochlear hair cells, and vestibular and low-frequency hearing deficits, indicating strong therapeutic potential (Lentz et al., 2013).

While malfunctioning splicing can be suppressed, recessive mutations in the protein-coding regions may not be as straightforward to target with SSOs. In parallel to the nusinersen clinical trial, exon-skipping SSOs have been developed to treat Duchenne muscular dystrophy (DMD). DMD is an X-linked progressive muscle-wasting disease caused by loss-of-function mutations in the DMD/dystrophin gene. The dystrophin protein has 24 repeated spectrin-like domains, and truncated dystrophin proteins with fewer spectrin-like repeats were found in patients who showed much milder symptoms (England et al., 1990). Human genetics studies indicate that bypassing exons in the middle of dystrophin while preserving its N- and C-terminal domains can be beneficial (Matsuo, 1996). About half of DMD patients have deletion mutations in a hotspot region between exons 45–55 (Duan et al., 2021). Multiple SSOs have been successfully developed to skip exons such as 51, 53, or 45 (Exondys 51, Vyondys 53, and Amondys 45) to correct translational reading frames and produce partially functional dystrophin proteins (Roberts et al., 2023) (Figure 1B).

Exon-skipping SSOs have been explored for targeting other diseases, such as correcting the reading frame in *CLN3* Batten’s disease (below), bypassing an inframe *CEP290* exon 41 that carries pathogenic mutations for Jobert syndrome (Ramsbottom et al., 2018), and suppressing a cryptic splice site in *CFTR* cystic fibrosis (Michaels et al., 2020). The Hastings group developed an exon-skipping strategy in mice to target a mutant allele that causes CLN3, a form of Batten’s disease (Centa et al., 2020). Patients carrying recessive *CLN3* mutations experience disease onset in early childhood and typically decease by 20–30 years of age (IBDC, 1995). A substantial portion of patients are affected by a deletion spanning exons 7 and 8 (Δex78), leading to a shift of the translational frame. SSOs have been reported to correct the reading frame by skipping exon 5 in *cis* (Δex578, Figure 1B). The SSO has been reported to robustly induce exon 5 skipping and improve motor coordination and survival in a *Cln3* (Δex78) mouse model. The research group further created a *Cln3* (Δex578) genetic model and showed that deleting exon 5 on top of Δex78 was beneficial in mice (Centa et al., 2023). These works suggest a promising exon-skipping strategy for CLN3 (Δex78) Batten’s disease.

The frontier of personalized medicine leaped forward with the N = 1 study on a child affected by CLN7, another form of Batten’s disease (Kim et al., 2019). CLN7 is a late-infantile-onset lysosomal storage disorder, and affected children would experience early normal development followed by function declines of the nervous system that lead to vision loss, drug-resistant epilepsy, progressive cerebral and cerebellar atrophy, and premature death (Topcu et al., 2004). CLN7 is caused by recessive mutations in *MFSD8* (Siintola et al., 2007), but in the N = 1 case, clinical testing only identified one inherited *MFSD8* allele (Kim et al., 2019). The Yu lab performed whole genome sequencing and identified an SVA-transposon insertion in *MFSD8* intron 6, which promoted the inclusion of a cryptic 3′ splice site in SSOs targeting predicted splicing enhancers were screened to suppress abnormal splicing of *MFSD8* (Figure 1A). The lead oligo milasen, an 18-nt SSO with 2’MOE modification and a PS backbone, was effective in patient cells and tolerated in rodents. Milasen was applied to the patient under an expanded-access protocol approved by the FDA and modeled after nusinersen. The *N* = 1 trial was shown to reduce seizure frequency and duration, suggesting a beneficial effect (Kim et al., 2019). This work paved the path for expedited genetic diagnosis and individualized drug development.

Important questions remain in SSO-mediated treatment. Given the diverse nature of pathogenic mutations, how can we identify targetable variants and design effective SSOs? A recent in-depth study of ataxia-telangiectasia (A-T) (Kim et al., 2023) began to address this question. A-T is an autosomal recessive disorder caused by the loss of the *ATM* gene required for DNA damage response and cell cycle progression (Savitsky et al., 1995). A-T patients typically show progressive cerebellar degeneration with early symptoms of ataxia, increased chance for cancer, and telangiectasias. A significant fraction of causal variants for A-T have been reported to cause abnormal splicing patterns (Teraoka et al., 1999), and SSOs (morpholino ASOs) have been developed to correct *ATM* splice variants (Du et al., 2007). A recent study reported whole-genome sequencing analyses of 235 A-T patients and classified plausible causal mutations depending on their molecular nature and potential for SSO treatment (Kim et al., 2023). Combining transcriptomic analyses and computation predictions, the authors estimated that 9 and 6% of the A-T patients carry “probable” and “possible” variants amenable to SSO targeting, respectively. The authors developed SSOs for two mutations and initiated clinical studies in A-T patients before disease onset. Thus, thorough genetics analysis estimated the SSO-targetable ratio to 9–15% in patients affected by rare diseases like A-T (Kim et al., 2023).

### SSOs for dominant diseases

The variant- or exon-specific SSO strategies used in recessive disorders, such as suppressing cryptic splice sites and bypassing deleterious mutations in nonessential exons (Figures 1A,B), are also applicable to target the mutated alleles in dominant genetic disorders, especially for gain-of-function/activity alleles. Following the initial linkage analyses and cloning of inherited mutations, recent human genetics studies discovered widespread dominant mutations causal for neurodevelopmental disorders such as epilepsy and autism spectrum disorders (Helbig and Abou Tayoun, 2016, Satterstrom et al., 2020). For instance, over 1,400 *SCN1A* mutations have been reported as pathogenic in ClinVar (a public database to aggregate genetic variants and clinical findings), and a significant fraction of such mutations cause severe loss of function (frameshift, nonsense, splice site, and deletion). Furthermore, causal mutations for neurodevelopmental disorders have been reported in dozens to hundreds of genes. However, targeting such a vast number of mutated alleles using variant- or exon-specific SSOs presents a daunting task.

For haploinsufficient mutations, the healthy allele offers another layer of therapeutic potential. Increasing protein expression from the healthy allele can potentially establish a gene-specific instead of a variant- or exon-specific solution. In principle, this is achievable by boosting transcription, suppressing mRNA degradation, promoting translation, or suppressing protein degradation. Strategies suppressing naturally occurring non-productive isoforms, boosting translation by recruiting ribosomes, degrading naturally occurring antisense transcripts, and targeted de-ubiquitination have been explored to treat haploinsufficiency (Meng et al., 2015; Han et al., 2020; Kanner et al., 2020; Lim et al., 2020; Cao et al., 2023; Dawicki-McKenna et al., 2023; Yang et al., 2023).

Abnormal translation termination caused by premature codons (PTCs) triggers nonsense-mediated mRNA decay (NMD) in eukaryotes (Kurosaki et al., 2019). Interestingly, naturally occurring alternative splicing can trigger NMD (AS-NMD), and AS-NMD has been shown to autoregulate the master splicing factor SR proteins (Lewis et al., 2003; Lareau et al., 2007; Leclair et al., 2020). Recent studies have reported that AS-NMD developmentally regulates hundreds of genes in the brain (Zheng et al., 2012; Eom et al., 2013; Yan et al., 2015). Abnormally elevated AS-NMD in *SNRPB*, *FLNA*, and *SCN1A* by human mutations have been reported to cause cerebro–costo–mandibular syndrome (Lynch et al., 2014), structural brain malformation (Zhang et al., 2016), and epilepsy in humans (Carvill et al., 2018). Thus, the naturally occurring non-productive alternative splicing in disease-associated genes can be targetable switches for gene regulation.

If the gene of interest naturally expresses an alternative and non-productive isoform, converting the non-productive splice isoform to a functional form would be a promising approach to upregulate gene expression. The TANGO (targeted augmentation of nuclear gene output) method was reported in 2020, with a focus on *SCN1A* (Han et al., 2020; Lim et al., 2020). *De novo* loss-of-function mutations in *SCN1A* are leading causes of DEE, especially the Dravet syndrome, which is characterized by intractable febrile seizures. Human genetic studies showed that a fraction of *SCN1A* mRNA contains exon 20 N that triggers nonsense-mediated decay, and if the inclusion is abnormally increased by human mutations, it causes Dravet syndrome (Carvill et al., 2018). Lim et al. started by looking for non-productive alternative splicing in human disease-associated genes, screened SSOs in cultured cells, and showed the efficacy of two *SCN1A* ASOs in mice (Lim et al., 2020). Zhou et al. further showed an in-depth screening of *SCN1A* ASOs, their effect in upregulating mRNA and protein expression in mice, and their striking effects in rescuing lethality in a Dravet syndrome mouse model (Han et al., 2020). Clinical trials of the SSO in Dravet patients are ongoing and appear promising. These studies suggest that targeting the non-productive isoform can be a promising therapeutic approach.

*SYNGAP1* encodes the synaptic Ras GTPase-activating protein and is required for synaptic plasticity. Haploinsufficient *SYNGAP1* mutations are the leading causes of intellectual disability, infantile epilepsy, and other neurological symptoms (Hamdan et al., 2009). Transcriptomic analysis of the developing mouse and human brains uncovered alternative 3′ splice sites of *SYNGAP1* intron10 that lead to NMD (A3SS-NMD, Figure 1C) (Yang et al., 2023). PTBP1/2 proteins directly promote the A3SS-NMD and suppress SYNGAP1 protein expression (Yang et al., 2023). Deletion of the A3SS-NMD in mice lead to upregulated Syngap1 protein. Importantly, such upregulated protein significantly alleviated the LTP deficits in the hippocampus and the neuronal excitability phenotype in cortical neurons caused by a compound *Syngap1* knockout allele (Yang et al., 2023). We further screened SSOs in human iPSCs, and the lead SSO effectively increased the functional *SYNGAP1* isoform in iPSC-derived neurons and cerebral organoids (Yang et al., 2023). Interestingly, some of the lead *SYNGAP1* SSOs identified in independent studies overlap with each other (Lim et al., 2020; Dawicki-McKenna et al., 2023; Yang et al., 2023), indicating the existence of a splicing enhancer for the A3SS-NMD.

Timothy syndrome, caused by dominant mutations in *CACNA1C*, is a multi-organ disorder characterized by congenital heart disease, lethal arrhythmias, cognitive deficits, and autism (Splawski et al., 2004). One recurrent p.G460R mutation occurs in the mutually exclusive exon 8A, promotes the exon 8A inclusion over exon 8, and leads to the loss of voltage-dependent channel inactivation (Panagiotakos et al., 2019). While *CACNA1C* exon 8 gradually replaces exon 8A during neural development, it was speculated as beneficial if the mutated exon 8A switched to exon 8 early in patients (Figure 1B). Indeed, the lead SSO was shown to increase *CACNA1C* exon 8 inclusion and rescue delayed channel inactivation and interneuron migration defects in cortical organoids (Chen et al., 2024). Furthermore, the authors transplanted cortical organoids carrying the p.G460R (exon 8A) mutation to athymic rats and showed the SSO treatment rescued molecular and functional defects (Chen et al., 2024). This study indicates that switching functionally equivalent but mutually exclusive exons can bypass deleterious effects and demonstrates the application of a human organoid-rat chimeric system.

### Rodent models

Cultured cell lines, patient-derived fibroblasts, human iPSCs, and iPSC-derived neural cultures provide valuable tools for SSO screens, and the *in vivo* testing of SSO toxicity in rodents has become an integral process before clinical studies. However, identifying SSOs that work effectively *in vivo* remains a major challenge. For the N = 1 or extremely rare life-threatening variants, the limited time frame would not allow the establishment of proper genetic models or the thorough *in vivo* testing of SSO efficacy. For SSOs that target a specific gene, an exon, or a recurrent allele, the *in vivo* studies would provide valuable insights. This is exemplified by the development of nusinersen, where the SMA mouse models provide crucial tools to determine the efficacy of SSOs at the molecular and physiological levels (Monani et al., 2000; Hua et al., 2011). More recently, the Dravet mouse model (*Scn1a* knockout) was instrumental in demonstrating the efficacy of the *SCN1A* SSO in upregulating protein expression and rescuing lethality (Miller et al., 2014; Han et al., 2020). While the *SCN1A* lead SSO sequence is conserved and can be conveniently tested in mice, this would not necessarily be true for many other targets and SSOs. Mice carrying human genes of interest, through either BAC transgenic or humanized gene replacement, would be helpful tools to facilitate SSO studies. Recently, athymic rats carrying transplanted human cortical organoids have been reported as a new chimeric model to test the efficacy of SSOs (Chen et al., 2024).

In addition to testing SSOs in models of human diseases, the feasibility of SSO strategies can also be genetically tested for the desired splicing changes. This has been demonstrated by genetically deleting exon 5 in the *CLN3* (Δex78) Batten’s disease model, where the *CLN3* (Δex578) allele has been shown to restore the reading frame and suppress the sensorimotor deficits (Centa et al., 2023) (Figure 1B, bottom). The heterozygous deletion of *Syngap1* A3SS-NMD has been recently shown to rescue haploinsufficiency in mice (Yang et al., 2023) (Figure 1C). These mouse genetic studies are critical to addressing questions that are otherwise hard to tackle: (1) Can the exon-skipping or NMD-suppression strategies yield the desired molecular and physiological outcome. For instance, when and how much protein upregulation can be achieved *in vivo* when the NMD exon is completely blocked. (2) Whether the splicing manipulation is deleterious for animal development. For the exon-skipping strategy, it is essential to know that the truncated protein would not gain toxicity or have more harmful effects than the otherwise loss-of-function allele. To treat haploinsufficiency by suppressing AS-NMD, it is crucial to understand the developmental function of the AS-NMD exons, which can be essential for brain development and functions. For example, deletion of the *Bak1* AS-NMD exon in mice induces abnormal neuronal loss and perinatal lethality (Lin et al., 2020). Homozygous deletion of A3SS-NMD exon in mouse Syngap1 led to deficits in long-term potentiation (Yang et al., 2023). Furthermore, CRISPR screens in cell lines have reported that AS-NMD exons can modulate cell proliferation and survival (Thomas et al., 2020). Thus, AS-NMD exons can be essential, and completely blocking AS-NMD may have undesired consequences. (3) Whether the genetic manipulation, mimicking the maximum effect of SSO treatment, can rescue or alleviate phenotypes in mouse models of human diseases.

### Outlook for SSO therapy

For developmental and progressive disorders, it is important to have an early genetic diagnosis for targeted therapy. The unprecedented identification of causal variants with exome, genome, and transcriptome analyses has set the stage for precision medicine. Genetic diagnosis takes only days to weeks and saves precious time for therapeutic development. The flexible yet specific targeting by SSOs and the clinically proven chemistry make it possible to target a particular gene, an exon, or even a unique mutation. This is achieved by suppressing cryptic splice sites, skipping specific exons, or boosting gene expression by redirecting naturally occurring alternative splicing. In addition to early-onset neurological disorders, SSOs have also been designed to target models of aging and neurodegenerative disorders (Chang et al., 2018; Korecka et al., 2019; Nikom and Zheng, 2023; Preussner et al., 2023). Furthermore, splice-modulatory small molecules are rising to treat neurological disorders such as SMA and Huntington’s disease (Palacino et al., 2015; Ratni et al., 2018; Bhattacharyya et al., 2021; Tang et al., 2021; Krach et al., 2022).

Naturally occurring alternative splicing events are potentially amenable to treating neurological disorders through different mechanisms: (1) Redirecting alternative splicing to promote the “healthier” allele. This has been demonstrated by nusinersen, which promotes SMN2 exon7 inclusion to make a stable protein. Most alternative exons (SE, A5SS, A3SS, MXE) are inframe, and pathogenic mutations within such exons can be potentially bypassed by enhancing alternative exon usage. (2) Treating haploinsufficiency by converting unproductive isoforms to functional forms. Suppression of *Scn1a* exon20N-NMD and *Syngap1* A3SS-NMD has been shown to alleviate haploinsufficiency in mice (Han et al., 2020; Yang et al., 2023). In *SCN1A*, *FLNA*, and *SNRPB* cases, deleterious mutations have been reported to increase AS-NMD and cause neurodevelopmental disorders (Lynch et al., 2014; Zhang et al., 2016; Carvill et al., 2018). Such human mutations may provide insights into how AS-NMD exons are regulated. In addition to AS-NMD, retained introns can be dynamically regulated and frequently prevent the host transcript from making functional proteins (Braunschweig et al., 2014; Mauger et al., 2016). Promoting intron excision may be another way to boost protein expression. Recent studies of nascent RNAs led to the estimation that ~15% of human protein-coding transcripts are degraded through AS-NMD, suggesting a large space for gene regulation (Fair et al., 2023). Deeper transcriptomic analyses and a better understanding of the splicing code will provide new insights into splice isoform regulation and enhance the discovery of SSO targets (Gandal et al., 2018; Li et al., 2018; Bao et al., 2019; Fair et al., 2023).

The gene- and exon-specific SSOs can be applied to conceivably many patients carrying mutations in the same gene or exon, and such SSOs have been going through clinical trials to determine their toxicity and efficacy. In contrast, variant-specific SSOs are enthusiastically pursued for personalized medicine or treating extremely rare cases (Kim et al., 2019; Crooke, 2022; Aartsma-Rus et al., 2023). Such N = 1 therapy presents new challenges and necessitates new guidelines for the SSO design and preclinical testing. An emerging question is what diseases, genes, and pathogenic variants are treatable by SSOs or ASOs in general. SSOs have been estimated to target 9–15% of A-T patients (Kim et al., 2023) and a higher ratio for DMD patients (Bladen et al., 2015). A much broader group of genes and about half of the pathogenic variants have been considered druggable with ASOs and other gene-regulatory mechanisms (Mittal et al., 2022). The active research and collaborative efforts in the field are drawing a promising future for SSO therapy.

## Author contributions

XZ: Writing – original draft, Writing – review & editing.
